# Response to isolated limb perfusion and chemotherapy with epirubicin plus ifosfamide in a metastatic malignant ossifying fibromyxoid tumor

**DOI:** 10.1186/s13569-017-0086-2

**Published:** 2017-12-28

**Authors:** Salvatore Provenzano, Alessandra Raimondi, Rossella M. Bertulli, Vittoria Colia, Salvatore L. Renne, Paola Collini, Gianpaolo Dagrada, Dario Callegaro, Marco Fiore, Francesca G. Greco, Paolo G. Casali

**Affiliations:** 1 Adult Mesenchymal Tumor and Rare Cancer Medical Oncology Unit, Cancer Medicine Department, Fondazione IRCCS Istituto Nazionale dei Tumori, Via G. Venezian 1, 20133 Milan, Italy; 2 Soft Tissue and Bone Pathology, Histopathology and Pediatric Pathology Unit, Department of Diagnostic Pathology and Laboratory Medicine, Fondazione IRCCS Istituto Nazionale dei Tumori, Milan, Italy; 3 Melanoma and Sarcoma Unit, Department of Surgery, Fondazione IRCCS Istituto Nazionale Tumori, Milan, Italy; 4 Department of Radiology, Fondazione IRCCS Istituto Nazionale dei Tumori, Milan, Italy

**Keywords:** Ossifying fibromyxoid tumor, Soft tissue sarcoma, Chemotherapy, Epirubicin, Ifosfamide, Isolated limb perfusion

## Abstract

**Background:**

Ossifying fibromyxoid tumor (OFMT) is a rare soft tissue neoplasm of uncertain lineage and intermediate biological potential. It is more common in middle-aged men, usually arising from the deep tissues of the extremities. It is now established that it is a translocation related tumor, most often marked by translocation of PHF1 gene. Surgery is the mainstay of treatment and proves usually curative, although, in rarer cases the disease shows malignant features and tendency to recur both locally and at distant sites. In such cases, no standard treatment exists.

**Case presentation:**

We report on a case of malignant advanced OFMT of the hand with lung metastases responding to isolated limb perfusion with human recombinant tumor necrosis factor and melphalan and chemotherapy with epirubicin and ifosfamide.

**Conclusions:**

To our knowledge, this is the first report of activity of soft tissue sarcoma-oriented chemotherapy in advanced OFMT.

## Background

Ossifying fibromyxoid tumor (OFMT) is a rare soft tissue tumor, originally described by Enzinger and Weiss in 1989 [1], and currently classified among neoplasms of uncertain origin and intermediate-grade behavior in the last WHO soft tissue tumors classification [2].

It usually onsets in middle-age, although cases have been reported in patients 14–79 years old, more commonly in males than females. Most cases arise as small, painless, subcutaneous masses often attached to the underlying tendons, fascia or skeletal muscle generally located at the extremities. Primary tumors of the trunk or the retroperitoneum are rarer [2].

OFMT consists of lobules of uniform, round to fusiform-shaped cells arranged in nests and cords in a set of variable fibromyxoid stroma usually surrounded by an incomplete shell of metaplastic, hypocellular, lamellar bone. However, this shell may lack in the so-defined non-ossifying OFMT. Mitotic activity is usually poor, counting for less than 1 per 10 high-power fields (HPF). Rarely, OFMT shows hypercellularity and increased number of mitotic figures with deposition of tumor osteoid that may simulate an osteosarcoma [2]. These variants have been described as “atypical” or “malignant”, and have been associated to a higher tendency to recur locally or metastasize, and to a higher risk of disease-related death [3–6], comparable to other soft tissue sarcomas. Overall, the local recurrence rate after complete surgical excision ranges between 17 and 27% [3, 6]. Metastases have been described only in malignant forms, and, as in other soft tissue sarcomas, are more frequent in the lungs and the soft tissues. Given the usual intermediate-grade behavior, a long survival is possible even in metastatic patients [2, 6].

It is now established that OFMT is a translocation related tumor. Up to 85% of cases carry PHF1 gene translocation, without significant differences among typical, atypical and malignant forms [7, 8], suggesting a precise role of this translocation in oncogenesis, still unknown. A possible mechanism of action may be through an epigenetic effect. Actually, PHF1 has a role in the regulation of chromatin structure, interacting with EZH1, EZH2 and SUZ12 [8]. Obviously, seeking the translocation is useful in differential diagnosis with other soft tissue neoplasms. Novel fusions involving BCOR, BCORL1 and WWTR1 have been reported [9, 10].

Complete surgical resection is usually curative and is the mainstay of treatment in the localized setting. In contrast, no standard exists in metastatic patients.

We report on a case of recurrent, advanced OFMT of the hand with multiple, bilateral, synchronous, lung metastases responding to treatments commonly used for other soft tissue sarcomas.

## Case presentation

**Fig. 1:**
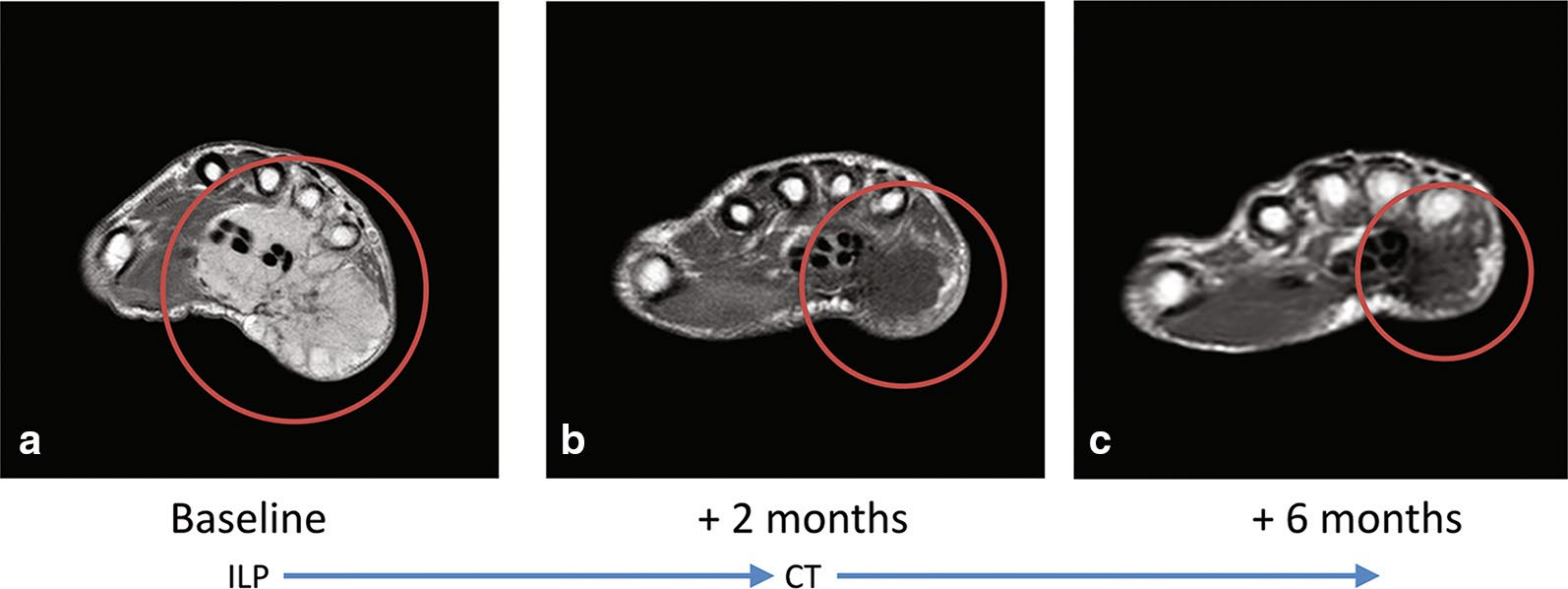
Axial contrast enhanced T1-weighted MRI. Pathologic tissue in the hypothenar eminence of the right hand, involving the flexor tendons and the spaces between 3rd, 4th and 5th metacarpal bones with intense and homogeneous contrast enhancement (**a**). Progressive size and contrast enhancement reduction after ILP (**b**), and chemotherapy (**c**)

**Fig. 2:**
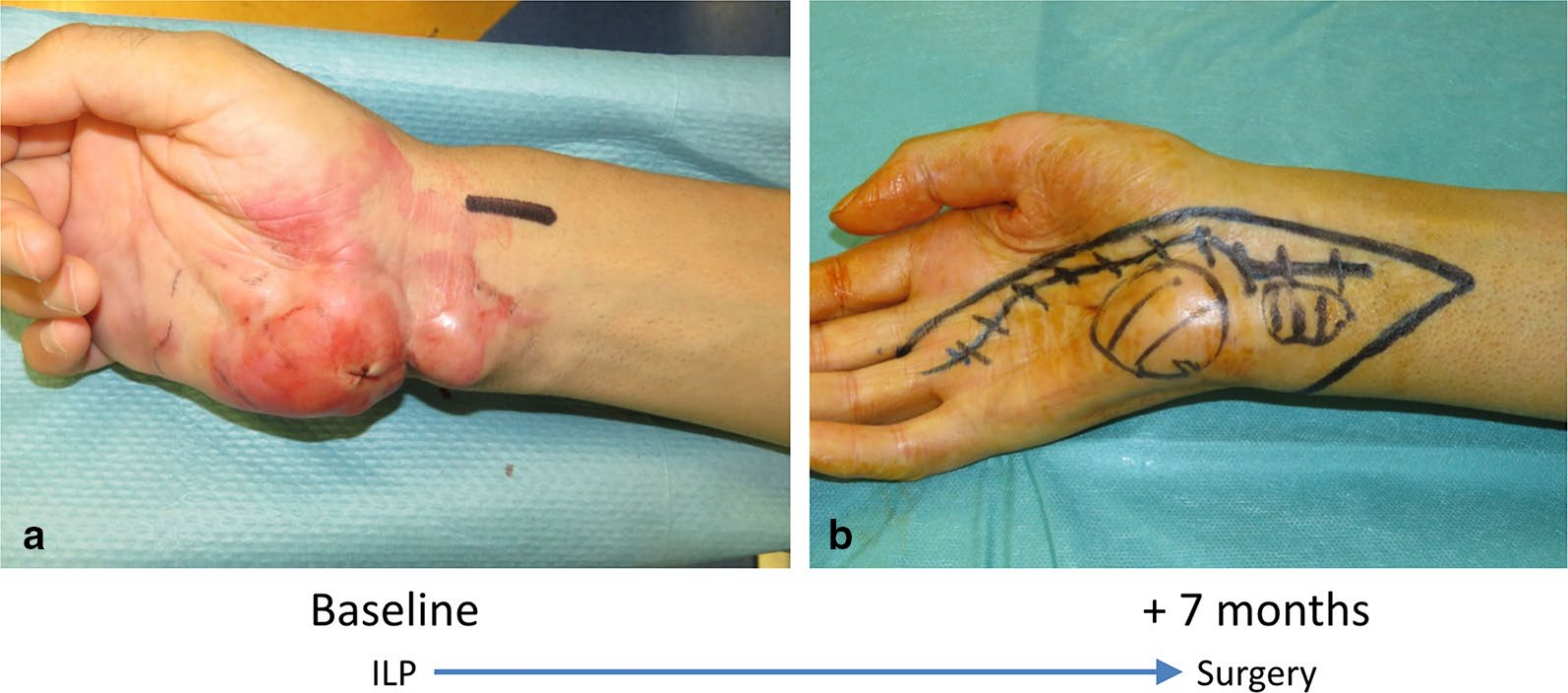
Picture of the patient’s hand. At presentation **a** the tumor involved widely the hypothenar eminence and medial aspect of the wrist. Seven months after ILP **b** the nodule in the wrist was hardly palpable while the nodule in the hand was smaller and softer

In June 2016, a 47-year-old man, in good performance status, presented at our Institution (Fondazione IRCCS Istituto Nazionale dei Tumori, Milan, Italy), with a multinodular relapse of a low-grade fibromyxoid sarcoma of the right hand (Figs. 1a, 2a), diagnosed in the past year upon surgery, performed elsewhere, of a 10-cm mass of the right palmar hand, originally infiltrating the tendons, vessels and nerves of the last two finger rays.

**Fig. 3:**
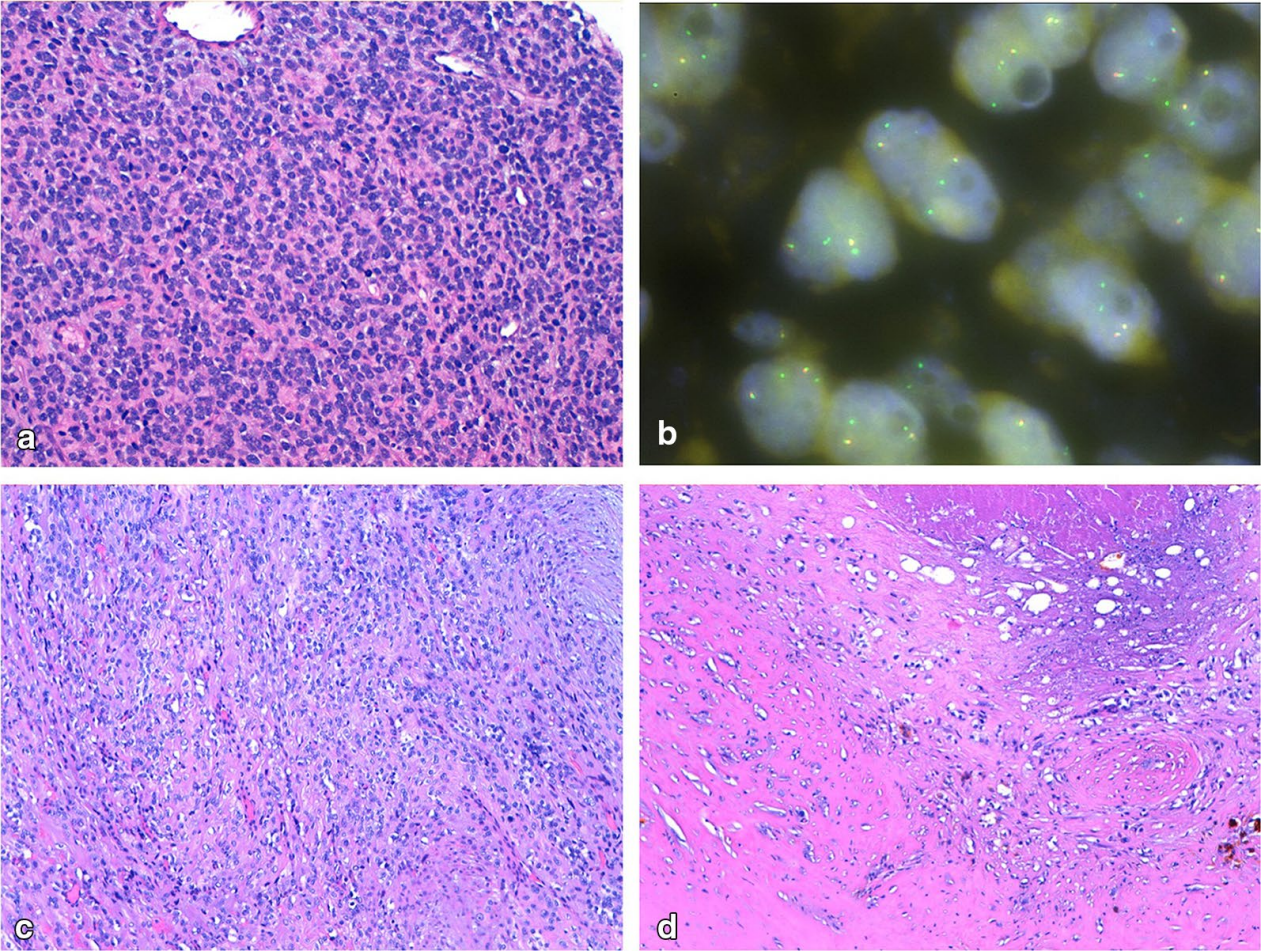
**a** Malignant OFMT pre-ILP biopsy, (HE, 40×). Densely packed cords of monomorphic cell with small round nuclei, high mitotic index. **b** Fluorescent in situ hybridization showing tetraploid cells showing unbalanced PHF1 gene translocation (green 5′). **c** Malignant OFMT post-ILP and chemotherapy, vital component (HE, 20×). The neoplasm is less densely cellulated and exhibits a variable morphology ranging from more rounded to spindle cell morphology. Mitotic activity is considerably reduced. **d** Malignant OFMT post-ILP and chemotherapy, non-vital component (HE, 20×). At the bottom of the image, a dense collagen sclerosis with haemosiderin deposition, ghost cells at the upper part, in between cell debris and isolated neoplastic cells with nuclei similarly to the viable component

The histopathological review showed a mesenchymal tumor arranged in a plexiform growth pattern, composed of small round cells, focal extracellular matrix in rose-shaped accumulation, and focal chondroid areas. The mitotic count was 14/10 HPF, necrosis was present (Fig. 3a). No ossifying shell was present. The diagnosis of malignant OFMT was confirmed by FISH test positive for PHF gene rearrangement (Fig. 3b).

**Fig. 4:**
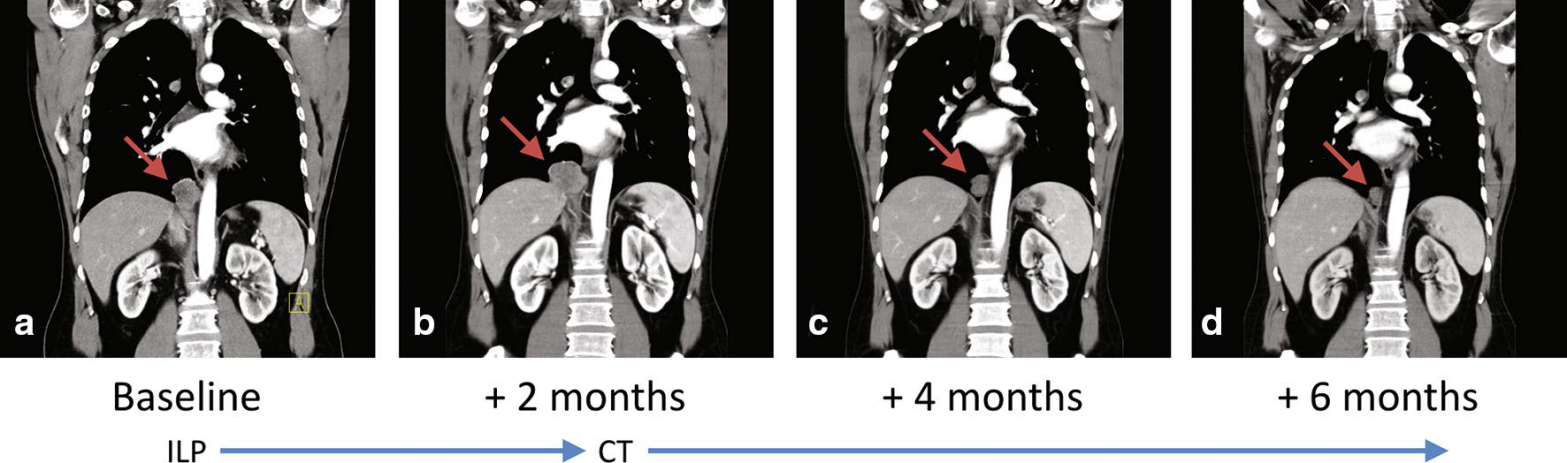
Coronal contrast enhanced CT scan. A right lung lower lobe metastases (arrow) at baseline (**a**), increasing after ILP (**b**), and progressively reducing after 3 (**b**) and 6 (**d**) cycles of chemotherapy with epirubicin and ifosfamide

A whole-body computerized tomography (CT) scan showed bilateral, multiple lung metastases (Fig. 4a).

At presentation, due to the extension of the disease to the third and fourth interdigital spaces and the distal forearm, an upfront surgery would have entailed a forearm amputation. To improve the chance of achieving a complete resection with a conservative procedure, in July 2016 the patient underwent isolated limb perfusion (ILP) of the upper right limb using recombinant human tumor necrosis factor (TNF) and Melphalan. The treatment was well tolerated and achieved an excellent clinical and radiological response, i.e. partial response (PR) according to RECIST (Fig. 1b).

In September 2016, when a new CT scan showed further progression of the lung metastases (Fig. 4b), and missing any evidence regarding the effectiveness of systemic treatment in this histotype, the patient underwent sarcoma-oriented chemotherapy with Epirubicin 105 mg/sqm and Ifosfamide 9000 mg/sqm + MESNA in 3 days every 3 weeks. Treatment related adverse events were febrile neutropenia, G3 anemia, G3-4 thrombocytopenia, and doses were reduced by 20% after the second cycle. We observed a PR both on the relapsed primary tumor and on the lung metastases after three and six cycles (Figs. 1c, 2b, 4c, d).

In February 2017, the Patient underwent complete surgery of the local residual disease, with amputation of the last two finger rays of his right hand. Therapy related changes accounted for 90% of neoplasm mostly composed of fibrosis with haemosiderin (85%) and focally by necrosis and ghost cells with loss of nuclear and cytoplasmic detail. Viable tumor was mostly located proximally, characterized by a heterogeneous morphology (Fig. 3c, d). Viable component was R1 on proximal resection margin, and palmar margin on soft tissue was R1 with therapy related changes.

In March and April 2017, the Patient underwent a 2-stage complete lung metastasectomy. Vital tumor component at the histopathological examination ranged between 85 and 100%. First post-treatment follow-up radiological evaluation performed in July 2017 resulted negative for persistent or relapsed disease.

## Discussion and conclusions

OFMT is a rare soft tissue neoplasm usually characterized by an intermediate-grade behavior [2]. Surgery is usually curative in typical variant, albeit local and distant relapses are reported in atypical and malignant subtypes [3–6]. There is no standard of treatment in case of metastatic disease.

We report on a case of advanced, malignant OFMT with a local relapse and bilateral lung metastases, treated with sarcoma-oriented treatment, and responding to ILP and chemotherapy with epirubicin and ifosfamide both on local and distant disease. Local tumor shrinkage enabled to perform a partially conservative surgery. The response of the lung metastases was strictly dependent on chemotherapy; indeed, the patient was progressing rapidly before treatment started, and achieved a good cytoreduction that led to considering bilateral metastasectomy.

ILP with TNF and Melphalan is a procedure that, in the setting of limb-threatening extremity STS, aims at improving the chance of performing a conservative resection. In short, it consists of infusion of high-dose TNF and melphalan directly in the affected limb under local hyperthermia by extracorporeal circulation. Complete pathological response after TNF-ILP has been described in up to half of cases. Moreover, TNF-ILP leads to distinct response patterns with devitalization of tumor margins; close or positive margins at critical structures thus may become acceptable. This may lead to conservative resections where amputation was planned. In different cohort studies, tumor resection and limb salvage in pre-planned amputations were achieved from 76 to 96% [11].

Anthracycline-based (doxorubicin or epirubicin) chemotherapy combinations have been widely used as first line treatment of metastatic soft tissue sarcomas [12]. The benefit of multi-agent compared to single agent anthracycline-based chemotherapy for advanced soft tissue sarcomas remains controversial. In most randomized prospective trials, combination regimens were associated with higher response rates (27–46%), despite a non-significant overall survival benefit [13, 14]. Indeed, it is essential to consider the goal of therapy in definition of the treatment plan. Sequential administration of active single agents may maximize the duration of disease control and reduce treatment-associated toxicities. However, upfront combination chemotherapy may be of benefit for selected patients, e.g. those with a high tumor burden requiring a prompt tumor shrinkage or those with a fast-growing disease: in these cases, the need of obtaining an objective response may justify the increased toxicity of drug combination.

Of course, it is difficult to conduct prospective trials in such a rare histotype, and even collecting clinical evidences from retrospective series becomes problematic, especially in the setting of orphan diseases. In fact, all published series of patients with OFMT focus on histopathological features and natural history.

Enzinger et al. [1] originally described the first 59 cases, with 11 patients experiencing a local relapse and 1 distant progression. Folpe et al. [6] analyzed a series of 70 patients and proposed the classification in “typical”, “atypical” and “malignant” types upon cellularity, nuclear atypia and mitotic index. In particular, tumors with a high nuclear grade and mitosis > 2/50 HPF were associated to a significant risk of developing distant metastases, and were labelled as “malignant” forms (6/10 patients with these features developed distant metastases, but only 1/16 with atypical OFMT, and 1/25 with a typical type). Miettinen et al. [3] associated mitosis > 2/50 HPF to a higher risk of developing a local relapse, but they concluded for a poor metastatic potential. However, their consideration may be limited from the high number of patients lost to follow-up. Graham et al. [15] observed 3 distant progressions among 15 patients with malignant OFMT, and none among 5 atypical and 26 typical type cases. Retrospective series on the natural history coming from the biggest series are summarized in Table 1. In all series, no data on the activity of chemotherapy have been reported [1, 3–6, 15].

**Table 1:** Histopathological and clinical features in published series of patients with OFMT

Study	Number of patients (total/FU)	Histological subtype (T, A, M)	Median follow-up (months)	Follow-up information	Outcome
Enzinger et al. [1]	59/41	N/A	N/A	11 LR 1 DM	3 DoC
Folpe et al. [6]	70/51	N/A	36	9 LR 8 DM	41 NED 6 ED 4 DoD 1 DoC
Miettinen et al. [3]	104/41	N/A	156	9 LR	32 UNK 16 DoC 5 NED
Graham et al. [15]	46/27	T = 26 A = 5 M = 15	55	2 LR (M) 3 DM (M)	3 DoD
Atanaskova Mesinkova et al. [4]	26/16	T = 8 A = 13 M = 5	45	3 LR (M) 1 DM (M)	N/A
Kossivi Dantey et al. [5]	6/6	A = 1 M = 5	27	No events	1 DoC

*FU* follow-up, *T* typical, *A* atypical, *M* malignant, *N/A* not available, *LR* Local relapse, *DM* distant metastasis, *DoC* dead of other cause, *NED* not evident disease, *ED* evident disease, *DoD* dead of disease, *UNK* unknown

Although in a single case, but in a rare disease and in absence of any other evidence, we provide data of activity of soft tissue sarcoma-oriented treatment in advanced, malignant OFMT. As far as we know, this is the first report of activity of chemotherapy in this histotype. Even though these cases are extremely rare, we believe that reporting anecdotal evidence is worth as helping the clinician to manage these patients, providing clinical data supporting the medical decision and our comprehension of these diseases. In this sense, all attempts of data collection should be supported and encouraged.

## Abbreviations

OFMT: ossifying fibromyxoid tumor
WHO: World Health Organization
HPF: high power field
PHF1: PHD finger protein 1
EZH1: enhancer of zeste 1 polycomb repressive complex 2 subunit
EZH2: enhancer of zeste 2 polycomb repressive complex 2 subunit
SUZ12: SUZ12polycomb repressive complex 2 subunit
BCOR: BCL6 corepressor
BCORL1: BCL6 corepressor like 1
WWTR1: WW domain containing transcription regulator 1
CT: computerized tomography
ILP: isolated limb perfusion
TNF: tumor necrosis factor
PR: partial response
RECIST: response evaluation criteria in solid tumors
R1: cancer cells present microscopically at the resection margin
HE: hematoxylin/eosin
